# Rubella Virus Strain-Associated Differences in the Induction of Oxidative Stress Are Independent of Their Interferon Activation

**DOI:** 10.3390/v10100540

**Published:** 2018-10-03

**Authors:** Sarah Zobel, Mechthild Lorenz, Giada Frascaroli, Janik Böhnke, Nicole C. Bilz, Megan L. Stanifer, Steeve Boulant, Sandra Bergs, Uwe G. Liebert, Claudia Claus

**Affiliations:** 1 Institute of Virology, University of Leipzig, 04103 Leipzig, Germany; zobel.sarah@icloud.com (S.Z.); Mechthild.Lorenz@medizin.uni-leipzig.de (M.L.); janik.boehnke@gmail.com (J.B.); Christin.Emmrich@medizin.uni-leipzig.de (N.C.B.); Sandra.Bergs@medizin.uni-leipzig.de (S.B.); liebert@medizin.uni-leipzig.de (U.G.L.); 2 Leibniz Institute for Experimental Virology, Heinrich Pette Institute, 20251 Hamburg, Germany; giada.frascaroli@leibniz-hpi.de; 3 Schaller Research Group at CellNetworks, Department of Infectious Diseases, Virology, Heidelberg University Hospital, 69120 Heidelberg, Germany; m.stanifer@dkfz-heidelberg.de (M.L.S.); s.boulant@Dkfz-Heidelberg.de (S.B.); 4 Research Group “Cellular Polarity and Viral Infection” (F140), German Cancer Research Center (DKFZ), 69120 Heidelberg, Germany

**Keywords:** *N*-acetyl-l-cysteine, MitoTEMPO, caspase 3/7, apoptosis, interferon-stimulated gene, viperin, ISG15, macrophage

## Abstract

Rubella virus (RV) infection impacts cellular metabolic activity in a complex manner with strain-specific nutritional requirements. Here we addressed whether this differential metabolic influence was associated with differences in oxidative stress induction and subsequently with innate immune response activation. The low passaged clinical isolates of RV examined in this study induced oxidative stress as validated through generation of the reactive oxygen species (ROS) cytoplasmic hydrogen peroxide and mitochondrial superoxide. The addition of the cytoplasmic and mitochondrial ROS scavengers *N*-acetyl-l-cysteine and MitoTEMPO, respectively, reduced RV-associated cytopathogenicity and caspase activation. While the degree of oxidative stress induction varied among RV clinical isolates, the level of innate immune response and interferon-stimulated gene activation was comparable. The type III IFNs were highly upregulated in all cell culture systems tested. However, only pre-stimulation with IFN β slightly reduced RV replication indicating that RV appears to have evolved the ability to counteract innate immune response mechanisms. Through the data presented, we showed that the ability of RV to induce oxidative stress was independent of its capacity to stimulate and counteract the intrinsic innate immune response.

## 1. Introduction

Infection with teratogenic rubella virus (RV) during the first trimester of pregnancy can cause severe defects during embryonic development. At later time points the risk for rubella embryopathy and the severity of its outcome are declining [1,2]. Despite the availability of a successful vaccine, outbreaks still occur, even in regions with a sufficient vaccine coverage [3,4]. RV establishes a persistent infection in various cell lines and is considered a rather slow-replicating virus with a low level of cytopathogenic effect (CPE) induction. On susceptible cell lines such as Vero (African green monkey kidney cell line), the rabbit kidney cell line RK13 [5] and the human adult lung fibroblast cell line Hs888Lu [6] cytopathogenic alterations are noted through cell rounding and cell detachment, which differ among RV clinical isolates. During isolation of RV from clinical specimens in most cases no sign of CPE development is noted, even on susceptible cell lines and despite passaging of the infected cultures [7].

The rather slow replication cycle of RV is characterized by a comparatively long eclipse period of 12 h [8] and appears to be supported by its complex interaction with cellular metabolism. While members of the closely related alphaviruses require glycolysis [9], RV alters the entire bio-energetic state of its host cells and increases glycolytic and respiratory activity [10]. Although this ability was present among different RV low passaged clinical isolates, some RV strains require exogenous glutamine to initiate metabolic alterations [10]. The close association of RV with the mitochondria is emphasized by an increased activity of the mitochondrial respiratory chain complex II [11]. Furthermore, the RV capsid protein localizes to the mitochondria [12], which appears to be connected with the modulation of mitochondria-based apoptotic pathways [13], including opening of the mitochondrial permeability transition pore [14]. Mitochondria, namely the electron transport chain, are an important source of reactive oxygen species (ROS) and represent a target for viral modification [15]. The level of ROS is indicative of the oxidative stress in a cell and ROS is found as three major species: hydrogen peroxide (H_2_O_2_), superoxide anion (O_2_^−^) and hydroxyl radical (HO). The level of ROS present inside a cell influences various cellular functions, including innate immune response pathways [15]. During Dengue virus infection, the activation of innate immune responses, as assessed by the expression level of antiviral and inflammatory genes and cytokine release assays, is positively correlated with the generated ROS level [16]. Oxidative stress can influence various cellular signalling pathways, including interferon (IFN)-based antiviral responses. Pre-treatment of the human hepatocellular carcinoma cell line Huh-7 with the oxidative stress generator H_2_O_2_ reduced the activation of the JAK/STAT (Janus kinase-signal transducer and activator of transcription) pathway by IFN α [17].

Innate immune responses constitute the first line of defence against incoming pathogens. They are activated by pattern recognition receptors, with toll-like receptor 3 (TLR3), retinoic acid-inducible gene I (RIG-I) and the melanoma differentiation-associated antigen 5 (MDA5) being the most important ones for RNA virus infections [18]. Sensing events are followed by production of IFNs, namely type I (IFN α and β), type II (IFN γ, restricted to specialized immune cells) and type III (IFN λ). After the binding of IFNs to their respective receptors, a panel of interferon-stimulated genes (ISGs) are induced which lead to the activation of an antiviral state in both the infected and neighbouring cells by multiple mechanisms [19].

Through this study, we addressed two main points: first, are RV strain-specific differences in metabolic alterations reflected by the level of ROS induction, which in turn could influence the induction of an innate immune response as assessed by activation of IFNs and expression of ISGs. Second, do RV strains induce the same type of IFNs and ISGs at comparable levels. Besides Vero cells, a well-characterized cell type for RV infection, epithelial human alveolar A549 cells, primary monocyte-derived human macrophages (Mφ) and human umbilical vein cells (HUVECs) were used. As representatives of the airway epithelium, the A549 cells mimic the natural, primary site of systemic RV infection. Since alveolar macrophages have been stained positive for RV antigen in tissue samples from fatal congenital rubella syndrome (CRS) cases [20], human M1-Mφ, that resemble human alveolar primary macrophages [21] and the M2-Mφ have been investigated. Finally, the endothelial cells HUVEC were chosen to analyse the effects of RV infection on the blood vessels [20].

On Vero cells the generation of ROS by RV strains was positively correlated with their ability to induce CPE. However, no association of oxidative stress induction with activation of IFN response was detected. All RV strains analysed in this study induced a profound type III IFN response but were especially sensitive against type I IFN, namely IFN β. This indicates that RV has evolved an efficient means to escape from IFN response mechanisms, which could support the RV strain-independent ability to replicate in M1- and M2-Mφ.

## 2. Material and Methods

### 2.1. Cell Lines and Cultivation

The African green monkey kidney cell line Vero (CCL-81) and the human lung adenocarcinoma cell line A549 were obtained from American Type Culture Collection (ATCC) and cultivated in Dulbecco’s modified Eagle’s medium (DMEM) with high glucose (25 mM), GlutaMAX (Gibco, Thermo Fisher Scientific, Darmstadt, Germany), 10% foetal calf serum (FCS) and the antibiotics penicillin (100 IU/mL) and streptomycin (100 µg/mL) at 37 °C in a humidified incubator with 5% CO_2_ atmosphere. Additionally, HEPES at a final concentration of 1mM was added to the cultivation medium of A549 cells. For maintenance of HUVEC (pooled population, PromoCell, Heidelberg, Germany) endothelial cell basal medium 2 was used without the addition of antibiotics. Primary human M1- and M2-macrophages (Mφ) were generated as previously described [22]. Briefly, primary monocytes were magnetically isolated (Monocyte Isolation Kit II, Miltenyi Biotec, Bergisch Gladbach, Germany) starting from peripheral blood mononuclear cells (PBMC) obtained from buffy-coats of healthy blood donors. 3 × 10^6^ per mL monocytes were seeded in culture medium (RPMI 1640 supplemented with 10% FCS, 2 mM L-glutamine (Biochrom AG, Berlin, Germany)), 100 U per mL penicillin and 100 U per mL streptomycin in hydrophobic Lumox^®^ dishes and differentiated into pro-inflammatory M1- or anti-inflammatory M2-Mφ by incubation for seven days in the presence of 100 ng per mL recombinant human granulocyte-macrophage colony-stimulating factor (rhGM-CSF) or rhM-CSF (R&D Systems, Minneapolis, MN, USA), respectively.

### 2.2. Ethic Statement

Buffy-coats were purchased from the Transfusion Centre of the Ulm University Medical Centre (IRB granted to the Institut für Klinische Transfusionsmedizin und Immungenetik Ulm GmbH, Ulm, Germany) and were obtained from anonymized healthy blood donors. All blood donors gave written informed consent to authorize the use of their blood for medical, pharmaceutical and research purposes.

### 2.3. Virus Strains and Virus Infection

RV stock virus was generated on Vero cells. Only a limited number of passages was used, as RV could obtain cytopathogenic properties during multiple passages [7]. To achieve a high rate of infection, Vero, A549 and M1- and M2-Mφ were infected with an MOI of 5, while HUVEC were infected with an MOI of 10 [8]. The following RV strains (genotypes are given in brackets) were used: in addition to the Vero cell-adapted laboratory strain F-Therien three clinical isolates with the WHO identification RVi/Gdansk.POL/14.07_07-00426 (07-00426, genotype 1E), RVi/Prahova region.ROU/25.03_03-03703 (03-03703, genotype 1G) and RVi/Wuerzburg.DEU/47.11_12-00009 (Wb-12, genotype 2B). RV-infected Vero, A549 and M1- and M2-Mφ were incubated with virus for up to three days, while HUVEC infection was limited to 36 h.

### 2.4. RV Titre Determination

The CPE-positive Therien and Wb-12 RV strains were titred on Vero cells by standard plaque assay (titre expressed as plaque forming unit, PFU per mL). Titre of low cytopathogenic 03-03703 and 07-00426 was determined by a focus—forming assay (titre given as focus forming unit, FFU per mL) as described previously [23] with the exception that TrueBlue™ peroxidase was used as the substrate (Medac GmbH, Wedel, Germany).

### 2.5. Viral Genomic RNA Quantification

For assessment of intracellular viral genomic RNA TaqMan-based one-step reverse transcription quantitative PCR was performed after extraction of total RNA by MagNA Pure technology (Roche Life Sciences, Mannheim, Germany) as described [23]. Briefly, sense (RV_235.s, 5′-CTG CAC GAG ATY CAG GCC AAA CT-3′) and antisense (RV_419.as, 5′-ACG CAG ATC ACC TCC GCG GT-3′) primers were used together with TaqMan fluorogenic probe (RV_291TaqFAM, 6FAM-TCA AGA ACG CCG CCA CCT ACG AGC-BBQ) for amplification of a conserved region within the p90 gene.

### 2.6. Cellular mRNA Expression

Trizol reagent (Thermo Fisher Scientific) was used to extract total RNA from mock- and RV-infected cells at indicated time points. For the analysis of the mRNA expression level of cellular targets, 1.5 to 2 µg of total RNA were reverse transcribed with oligo(dT)_18_ primer and AMV reverse transcriptase (Promega, Mannheim, Germany) at 42 °C for 1 h, followed by 10 min at 70 °C. SYBR Green was used as PCR dye in quantitative real-time PCR (qRT-PCR) experiments based on carousel-based LightCycler 2.0 (Roche). For qRT-PCR assays, the GoTaq*^®^* qPCR master mix (Promega) was used with a 1:5 dilution of the cDNA samples together with 1 µg BSA. For normalization in the 2^−ΔΔ*C*t^ method the hypoxanthine guanine phosphoribosyl transferase (HPRT1) gene was employed. The mRNA expression level of selected genes was determined by the following primer pairs (forward/reverse) at the respective annealing temperature given in brackets:

HPRT1 TGACACTGGCAAAACAATGCA/GGTCCTTTTCACCAGCAAGCT (62 °C), [24];
SOD2 TGGCTTGGTTTCAATAAGGAA/AGCGTGCTCCCACACATCAAT (58 °C), (RTPrimerDB);
p21 CCTGTCACTGTCTTGTACCCT/GCGTTTGGAGTGGTAGAAATCT (52 °C), [25];
IFNβ1 GCCGCATTGACCATCTAT/GTCTCATTCCAGCCAGTG (60 °C);
IFNλ1 GCAGGTTCAAATCTCTGTCACC/AAGACAGGAGAGCTGCAACTC (60 °C);
IFNλ2/3 GCCACATAGCCCAGTTCAAG/TGGGAGAGGATATGGTGCAG (60 °C);
viperin GAGAGCCATTTCTTCAAGACC/CTATAATCCCTACACCACCTCC (60 °C);
IFIT1 AAAAGCCCACATTTGAGGTG/GAAATTCCTGAAACCGACCA (60 °C);
IFITM1 CCAAGGTCCACCGTGATTAAC/ACCAGTTCAAGAAGAGGGTGTT (56 °C), [26];
IFITM3 GATGTGGATCACGGTGGAC/AGATGCTCAAGGAGGAGCAC (55 °C);
ISG15 CTGTTCTGGCTGACCTTCG/GGCTTGAGGCCGTACTCC (56 °C), [26];

### 2.7. Cell-Based Assays

Cell permeable Hoechst bisbenzimide 33342 (Thermo Fisher Scientific) was included at 2 µM in all cell-based assays as a DNA counterstain.

### 2.8. Analysis of Apoptosis Induction

Alexa Fluor 488 Annexin together with propidium iodide (PI) were used to identify apoptotic and dead cells following manufacturer’s instructions for the Alexa Fluor^®^ 488 Annexin V/Dead cell Apoptosis Kit (Thermo Fisher Scientific). For assessment of caspase 3 and 7 activation the CellEvent caspase-3/7 Ready Probes^®^ reagent (Thermo Fisher Scientific) was used. The cleavage of the non-fluorescent DEVD substrate by these caspases results in fluorescent signals, which were monitored through fluorescence microscopy.

### 2.9. Detection of ROS and Application of ROS Scavengers

The previous assessment of intracellular ROS generation during RV infection [11] was extended by the use of two additional ROS-sensitive dyes. The compound CM-H_2_DCFDA (Thermo Fisher Scientific) is trapped intracellularly after its unspecific cleavage by esterase to 2′-7′ dichlorofluorescein (H_2_DCF). H_2_O_2_ as one of the most important ROS species was detected after oxidation of H_2_DCF to the highly fluorescent 2′-7′ dichlorodihydrofluorescein (DCF). CM-H_2_DCFDA was applied to cells at 5 µM in DMEM with 1% FCS and cells were washed with phosphate buffered saline (PBS) after an incubation of 10 min. Besides CM-H_2_DCFDA, MitoSOX™ Red mitochondrial superoxide indicator (Thermo Fisher Scientific) was applied at 5 µM in HBSS and cells were washed with HBSS after an incubation of 30 min. As positive controls for cellular ROS generation, cells were incubated overnight (A549) or for 5 h (Vero) with 0.03% hydrogen peroxide (H_2_O_2_) or with 30 µM oligomycin. As a positive control for mitochondrial superoxide, antimycin A (20 and 200 µM) was applied for 30 min. As cytoplasmic ROS scavenger *N*-acetyl-l-cysteine (NAC; Merck, Sigma-Aldrich, Taufkirchen, Germany) was applied at 5 mM to RV-infected Vero cells at 2 days post-infection (dpi) for further incubation until 3 dpi. In the case of the positive controls, NAC was added 16 h before induction of ROS through addition of H_2_O_2_ or oligomycin. Accordingly, the mitochondrial superoxide scavenger MitoTEMPO (Merck, Sigma-Aldrich) was added at a concentration of 500 µM to RV-infected Vero cells at 2 dpi for further incubation until 3 dpi.

### 2.10. IFN Response and Stimulation Assays

The secretion of IFN α, β, γ and λ was determined by the LEGENDplex human type 1/2/3 IFN panel (BioLegend, San Diego, CA, USA). Experiments were carried out on an Accuri C6 flow cytometer (BD Bioscience, Heidelberg, Germany) according to manufacturer’s instructions. As a positive control, transfections were carried out with 1 µg of the double-stranded (ds) RNA analogue polyinosinic-polycytidylic acid (poly I:C; Santa Cruz Biotechnology, Heidelberg, Germany) per well of a six well plate using lipofectamine2000 (Thermo Fisher Scientific) as transfection reagent. For stimulation of A549 cells with human IFN λ a mix of IFN λ1 and 2 (interleukin-29/IL-29, interleukin-28A/IL-28A, Peprotech, Hamburg, Germany) and IFN λ3 (interleukin-28B/IL-28B; Novoprotein, Summit, NJ, USA) at 66 ng per mL of each IFN λ and IFN β (Peprotech) at 100 ng per µL was used. A549 cells were incubated with IFNs for 48 h and were plated 24 h prior to infection with RV in the continued presence of the respective IFN.

### 2.11. Immunofluorescence Analysis

Cells grown on glass coverslips were fixed with paraformaldehyde (2% *w*/*v*) for 10 min and after washing with PBS, cells were permeabilized with ice cold methanol at −20 °C for 5 min. Thereafter samples were washed with PBS and incubated with primary monoclonal antibody Mab to E1 protein (Viral Antigens Incorporation, Memphis, TN, USA) at a dilution of 1:200 for 1 h in a humid chamber at 37 °C. After three washes with PBS the secondary antibody was applied for 45 min under same conditions. Then the DNA counterstain Hoechst bisbenzimide 33342 was applied and coverslips were mounted in Fluoromount G (SouthernBiotech, Birmingham, AL, USA).

### 2.12. Western Blot Analysis

At 3 dpi mock- and RV-infected A549 cells were subjected to extraction of cytoplasmic and nuclear fractions by the NE-PER nuclear and cytoplasmic extraction (Thermo Fisher Scientific). To ensure equal gel loading of 40 µg per lane, total protein concentration was determined by standard Bradford assay. Only cytoplasmic fractions were subjected to SDS-page and electroblotting in transfer buffer composed of 20 mM Tris, 150 mM glycine and 20% methanol (*v*/*v*)). Membranes were blocked with 5% (*w*/*v*) non-fat dried milk powder and 0.1% Tween-20 in PBS. Thereafter overnight incubation of the PVDF membrane with a 1:1000 dilution of the anti-E1 antibody (MAB925, Merck Chemicals, Darmstadt, Germany) was performed at 4 °C. The incubation with horseradish peroxidase-conjugated anti-mouse and anti-rabbit secondary antibodies was followed by detection with a commercial ECL solution (Thermo Fisher Scientific) used for chemiluminescence. For data collection, the C-DiGit blot scanner (LI-COR Biosciences, Bad Homburg, Germany) was used followed by image adjustment with only slight alterations to brightness and contrast using Corel DRAW X7.

### 2.13. Statistical Analysis

Data in the diagrams are shown as means ± standard deviation (SD). The assay setup was based on three independent experiments unless otherwise stated. Statistical significance was calculated using Prism software (GraphPad Software, Inc., La Jolla, San Diego, CA, USA). Differences in virus titres were verified with an unpaired parametric two-tailed Welch’s *t* test, while normalized mRNA expression levels were analysed by a paired Student’s *t* test (consistent ratios of paired values). For comparison of two viruses unpaired Student’s *t* test was performed, whereas for more than two groups or samples with multiple experimental parameters, one-way analysis of variance (ANOVA), followed by Tukey’s post hoc analysis, was used. The level of significance is indicated in the diagrams by asterisks (*, *p* < 0.05; **, *p* < 0.01; ***, *p* < 0.001; ****, *p* < 0.0001).

## 3. Results

### 3.1. Infection of Vero Cells with RV Strains Results in Differential Induction of Apoptosis and Oxidative Stress

To represent the currently circulating genotypes 1E, 1G and 2B [27], the clinical isolates 07-00426, 03-03703 and Wb-12 were used for infection studies in addition to the laboratory Therien strain. In a previous study performed in Vero cells, the viral replication rate, virus-associated metabolic alterations and cytopathogenicity were characterized in each of these strains [10]. While Wb-12 was comparable to Therien, the isolates 03-03703 and 07-00426 exhibited a lower replication rate and cytopathogenic potential than Wb-12 and Therien [10]. Thereafter RV strains will be arranged due to their cytopathogenic potential in decreasing order, namely Therien, Wb-12, 03-03703 and 07-00426.

**Figure 1:**
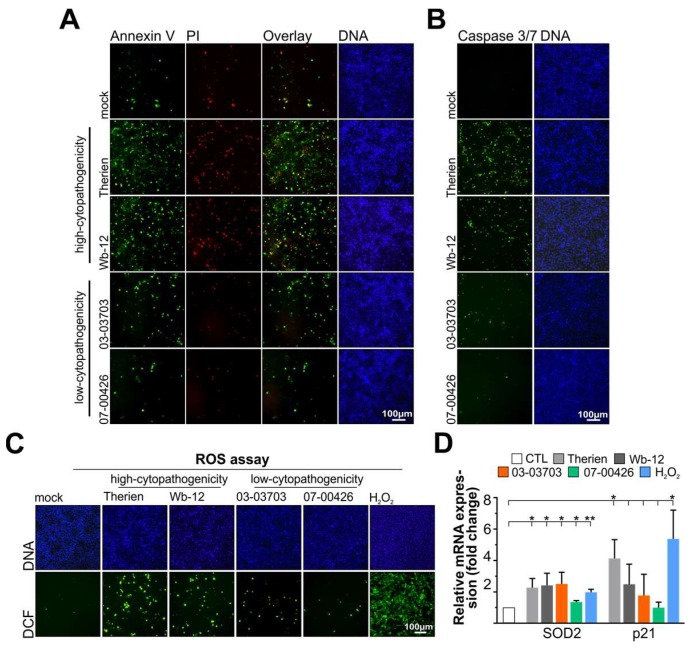
Comparative analysis of the capacity of different rubella virus (RV) strains to induce apoptosis and evoke an oxidative stress response in Vero cells. (**A**–**C**) At 3 days post-infection (dpi), RV strain-specific differences in apoptosis induction were assessed through fluorescence microscopy of (**A**) early (Annexin V-Alexa 488-positive cells) and late (Annexin V-Alexa 488- and propidium iodide (PI)-positive cells) apoptotic response and (**B**) activation of caspase 3 and 7 activity through monitoring nuclear accumulation of the fluorophore after DEVD cleavage; (**C**) Reactive oxygen species (ROS) generation was monitored at 3 dpi in Vero cells infected with indicated RV strains through fluorescence microscopy of the dichlorofluorescein (DCF) fluorescence; (**D**) The mRNA expression level of the mitochondrial antioxidant enzyme superoxide dismutase 2 (SOD2) and the oxidative stress-sensitive protein p21 was validated by quantitative real-time PCR (qRT-PCR). Hydrogen peroxide (H_2_O_2_) was used as positive control and applied for 5 h at a 1:5000 dilution. CTL, control. *, *p* < 0.05; **, *p* < 0.01.

To address whether these differences in metabolic alterations, infection rate and cytopathogenicity are also reflected in the virus-induced apoptotic and oxidative stress responses, cells were infected for three days allowing for CPE development after infection with the Therien and Wb-12 strains. Annexin V staining showed that Therien and Wb-12 induced a higher level of apoptosis than 03-03703 and 07-00426 (Figure 1A). This was complemented by a higher proportion of propidium iodide (PI)-positive cells, both necrotic cells (solely PI), or late apoptotic cells (dual Annexin V and PI), (Figure 1A). Additionally, a higher level of caspase 3/7 activity was detected for Therien and Wb-12 compared to 03-03703 and 07-00426 strains as shown by generation of the fluorescence signal of a cleaved DEVD peptide (Figure 1B). Additionally, the ROS-sensitive dye CM-H_2_DCFDA was applied to RV-infected Vero cells to detect cytoplasmic ROS, mainly hydrogen peroxide (H_2_O_2_). The dye CM-H_2_DCFDA is retained intracellularly after cleavage of its acetate groups by esterases to CM-H_2_DCF. In the presence of ROS, CM-H_2_DCF is oxidized to the fluorescent CM-DCF (DCF) as an indicator dye. Fluorescence microscopy revealed a higher number of DCF-positive cells for Therien and Wb-12 strains as compared to 03-03703 and 07-00426 (Figure 1C). Despite a nearly 100% infection rate at this time point as noted for Therien and Wb-12 strains [10], ROS production during RV infection can be considered as marginal compared to the positive control H_2_O_2_ (Figure 1C). At earlier time points (1 and 2 dpi) the number of DCF-positive cells detected in RV-infected cell populations was comparable to the mock control, indicating that ROS production exceeds cellular antioxidant countermeasures only at late time points of infection.

The analysis of oxidative stress generation with the ROS-sensitive dye CM-H_2_DCFDA was complemented by evaluating the mRNA expression level of the mitochondrial antioxidant enzyme superoxide dismutase 2 (SOD2) and p21^waf1/cip1^, which participates in the cellular oxidative stress response [28]. A slight but significant accumulation of SOD2 mRNA was detected for Vero cells after infection with all RV strains, which was comparable to the positive control H_2_O_2_ (Figure 1D). In contrast to SOD2, differences were noted among RV strains in the mRNA expression level of p21^waf1/cip1^. The highest level of induction was noted for Therien, which almost reached the level of the positive control H_2_O_2_ (Figure 1D). Collectively our data indicates that RV strains differ in their level of apoptosis and oxidative stress induction, which was positively correlated with their level of cytopathogenicity.

### 3.2. The ROS Scavenger NAC Reduces RV Cytopathogenicity

**Figure 2:**
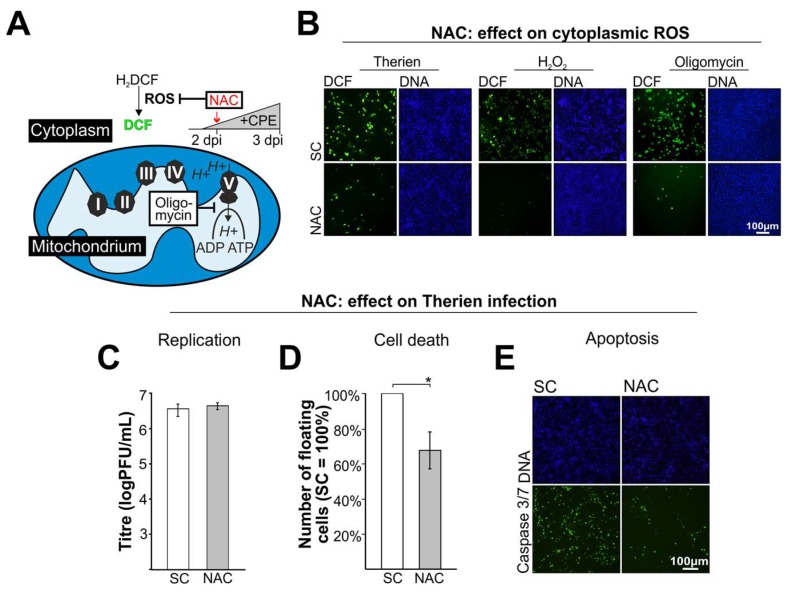
The ROS scavenger *N*-acetyl-l-cysteine (NAC) reduces RV-associated cytopathogenicity including induction of apoptosis. (**A**) Schematic overview of the application of NAC to RV-infected Vero cells at 2 dpi before the onset of cytopathogenic effect (CPE). The effect on ROS generation was detected by conversion of the non-fluorescent H_2_DCF to the fluorescent DCF; (**B**) The cytoplasmic ROS scavenger NAC was applied at 2 dpi to Vero cells infected with the cytopathogenic RV strain Therien. As positive controls, H_2_O_2_ and the ATP synthase inhibitor oligomycin were used. The effect on ROS generation was monitored through microscopic analysis of DCF fluorescence; (**C**–**E**) The effect of NAC on infection of Vero cells with RV Therien strain was assessed through (**C**) determination of virus titre by plaque assay; (**D**) rate of cell death as evaluated through the number of dead cells floating in the supernatant of Therien-infected cells; and (**E**) measurement of caspase 3/7 activation through microscopic evaluation of the fluorescence signal generated after cleavage of the DEVD peptide by caspase 3/7 from its conjugated fluorophore. SC, solvent control. *, *p* < 0.05.

As the Therien strain displayed a notable oxidative stress response it was used to study the effect of the ROS scavenger NAC on RV-associated oxidative stress response and cytopathogenicity. For cytoplasmic and mitochondrial ROS, H_2_O_2_ and oligomycin were used as positive controls, respectively. Cytoplasmic ROS generation in the presence or absence of NAC was monitored by fluorescence microscopy in Therien-infected and H_2_O_2_- and oligomycin-treated cells with the ROS-sensitive dye CM-H_2_DCFDA. The application of NAC at 5 mM (Figure 2A) reduced ROS levels in Therien-infected as well as positive control-treated Vero cells (Figure 2B). Comparable to the application of H_2_O_2_ and oligomycin, the ROS level was lower than the one observed for the solvent control (SC), (Figure 2B). While the application of NAC had no detectable effect on RV replication as assessed by generation of virus progeny (Figure 2C), Therien-associated cytopathogenicity was significantly reduced as determined by the reduction of the number of detached (floating) cells in the supernatant of RV-infected Vero cells [14,29] (Figure 2D). This reduction could be attributed to a lower activation of caspase 3/7 as detected after application of NAC (Figure 2E). In summary, NAC could scavenge RV-induced ROS generation and cytopathogenicity in a concentration dependent manner. The scavenging function of NAC on RV-infected cells was in accordance with its effect on the mitochondrial ROS inducer oligomycin, which indicates the presence of mitochondrial ROS in RV-infected Vero cells.

### 3.3. Nuclear Localization of the Superoxide Sensitive Dye MitoSOX Reveals Mitochondrial Dysfunction during RV Infection

**Figure 3:**
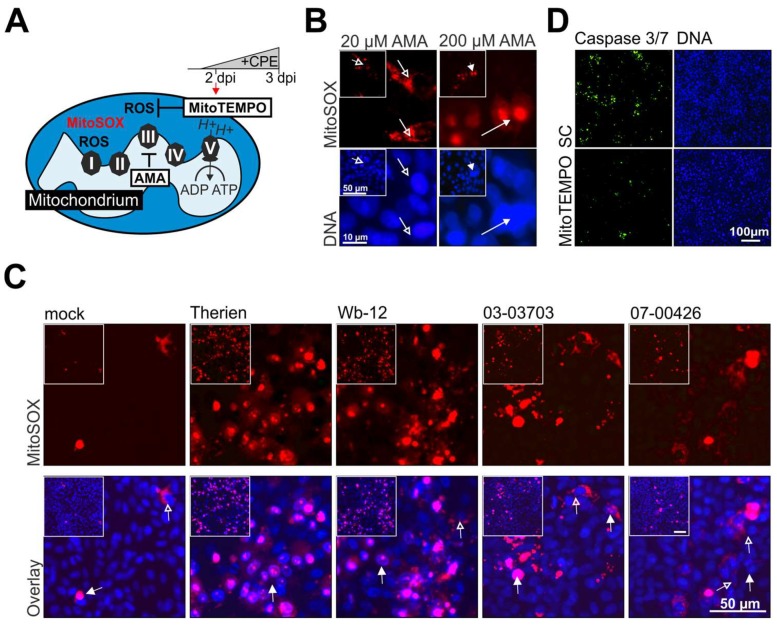
MitoSOX staining of RV-infected cells revealed the presence of mitochondrial superoxide and suggests mitochondrial dysfunction through its nuclear translocation. (**A**) Overview of the application scheme of superoxide detection by MitoSOX dye and scavenging by MitoTEMPO; (**B**) Depending on the applied concentration of antimycin A (AMA), MitoSOX stains a mitochondria-confined region in the cytoplasm (at 20 µM, open arrow) or within the nucleus (at 200 µM, closed arrow). The insets depict the corresponding microscopic field at a lower magnification; (**C**) At 3 dpi mock- and RV-infected Vero cells were incubated with MitoSOX and subjected to fluorescence microscopy. The inset provides the corresponding overview of the area depicted at higher magnification. The localization of the dye is indicated by open arrows for mitochondrial and closed arrows for nuclear localization; (**D**) At 2 dpi the mitochondrial antioxidant MitoTEMPO was added to Therien-infected Vero cells to assess its effect on apoptosis as compared to control cells in the absence of the scavenger (SC). Induction of apoptosis was monitored through activation of caspase 3/7. Activated caspase activity results in generation of a fluorescence signal after cleavage of the DEVD peptide from the fluorophore.

The observed increase in SOD2 mRNA expression (Figure 1D) and the differential effects of 5 mM NAC on RV-induced ROS generation point toward mitochondria-localized ROS during RV infection. Additionally, cytoplasmic H_2_O_2_ as detected during RV infection (Figure 1C) can also be converted from mitochondrial respiration-derived superoxide anion. To determine if this conversion takes places after RV infection, superoxide levels inside mitochondria were analysed by anion-sensitive dye MitoSOX Red (MitoSOX), (Figure 3A). As a positive control for mitochondrial ROS, antimycin A (AMA) was applied. As shown in Figure 3B, while the application of 20 µM AMA to Vero cells lead to an almost exclusive mitochondrial localization of the dye (highlighted by open arrows), at 200 µM AMA causes mitochondrial damage, which results in the release of the dye and its nuclear translocation (Figure 3B, closed arrows). This is in agreement with the reported nuclear confinement of MitoSOX dye after the loss of mitochondrial integrity [30]. Figure 3C shows that irrespective of the applied RV strain, at 3 dpi most of the MitoSOX dye is confined to the nucleus of RV-infected Vero cells. However, the number of MitoSOX-positive nuclei was higher for Therien and Wb-12 strains as compared to 03-03703 and 07-00426 strains. In addition to the nuclear localization, MitoSOX-staining was also detected inside mitochondria as depicted for some RV strains (Figure 3C, open arrows). We then assessed whether mitochondrial superoxide contributes to RV-induced apoptosis through application of the mitochondrial ROS scavenger MitoTEMPO to Therien-infected Vero cells. Similar to NAC, 2 dpi was chosen as application time point for MitoTEMPO as this is before the onset of CPE. Compared to the SC-treated population, MitoTEMPO reduced the number of caspase 3/7-positive cells as revealed by the fluorescence signal obtained after cleavage of the DEVD peptide (Figure 3D).

In conclusion, mitochondrial superoxide was generated during RV infection, which appeared to coincide with mitochondrial damage and thus nuclear translocation of MitoSOX. Accordingly, the application of the mitochondrial ROS scavenger MitoTEMPO reduced the level of RV-induced apoptosis.

### 3.4. On A549 Cells RV Strains Activate Type I and III IFNs and a Panel of IFN-Stimulated Genes in a Comparable Manner

**Figure 4:**
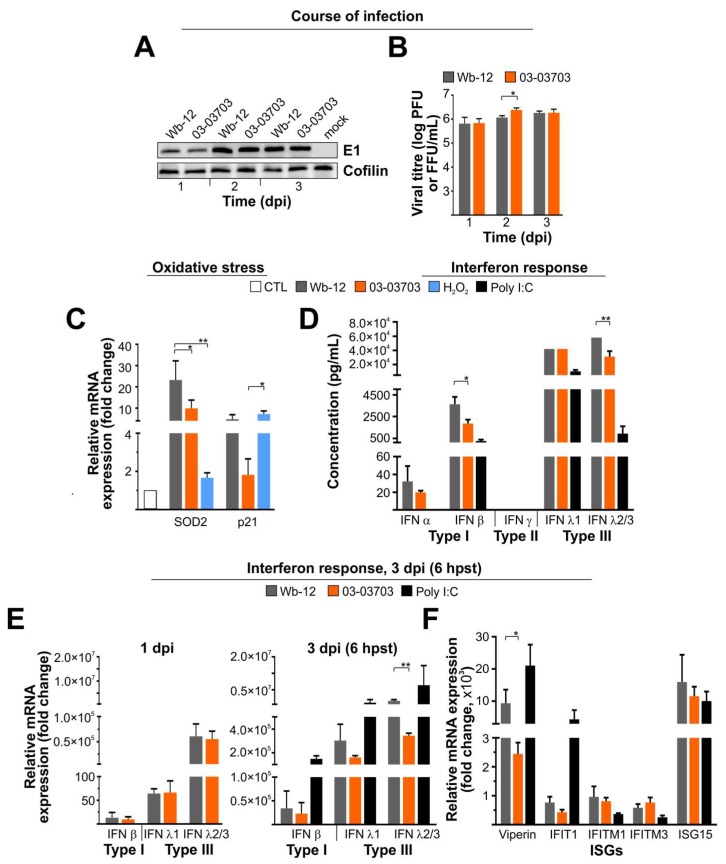
In contrast to the induction of oxidative stress, the RV-associated interferon (IFN) response on A549 cells was comparable among RV strains. (**A**) Western blot analysis of mock- and RV-infected A549 cells was performed at indicated time points with an antibody against RV E1 protein. The image shown is representative for three Western blot runs with independent samples; (**B**) Virus progeny generation was assessed by plaque (Wb-12) and focus forming assay (03-03703); (**C**) At 3 dpi the expression level of the antioxidant enzyme SOD2 and the oxidative stress-sensitive protein p21 was determined at mRNA expression level by qRT-PCR. H_2_O_2_ applied over night at a 1:5000 dilution was used as positive control; (**D**) The LEGENDplex IFN panel was used to obtain the IFN profile for Wb-12- and 03-03703-infected A549 cells at 3 dpi. The lack of error bars is due to the upper detection limit of the assay, which was present despite analysis of a 1:4 dilution of the supernatants collected from mock- and RV-infected A549 cells; (**E**) The type I and III IFNs mRNA expression level was determined by qRT-PCR for samples extracted from Wb-12- and 03-03703-infected A549 cells at 1 and 3 dpi; (**F**) Activation of IFN-stimulated genes (ISGs) was validated by qRT-PCR for Wb-12- and 03-03703-infected A549 cells (obtained at 3 dpi); (**E**,**F**) Expression level was normalized to hypoxanthine guanine phosphoribosyl transferase (HPRT1) and expression is given relative to the corresponding mock- or untreated control (CTL); (**D**–**F**) As a positive control, RNA extracted at 6 h post-transfection (hpst) from polyinosinic-polycytidylic acid (poly I:C)-transfected A549 cells was used. As a negative control mock-infected and lipofectamine2000-transfected samples were employed for RV infection and poly I:C transfection, respectively. *, *p* < 0.05; **, *p* < 0.01.

Due to the deletion of IFN genes, Vero cells lack the type I IFN system. Therefore, we employed A549 cells to analyse the IFN response induced by RV infection. Wb-12 and 03-03703 were used in subsequent infections as representative high and low CPE strains, respectively. In contrast to Vero cells, A549 cells showed comparable levels of viral E1 protein synthesis during Wb-12 and 03-03703 infection at all time points analysed (Figure 4A). Furthermore, there was no difference in viral titre observed between the two RV strains (Figure 4B). However, on A549 cells as compared to Vero cells, the Wb-12 strain induced a higher mRNA level of SOD2 and p21 than 03-03703 (Figure 4C) indicating a higher oxidative stress level upon Wb-12 infection as compared to 03-03703. Additionally, the mRNA expression level of SOD2 was significantly higher for Wb-12 as compared to 03-03703.

IFN response was addressed at 3 dpi through quantification of type I (IFN α and β), type II (IFN γ) and type III (λ1 and λ2/3) IFNs by LEGENDplex assay (Figure 4D). As a positive control, A549 cells were transfected with poly I:C and supernatants collected at 6 h post-transfection. Synthetic poly I:C is a well-characterized agonist of cytosolic pattern-recognition receptors MDA5 and RIG-I [31]. Among type I IFNs, a robust secretion was detected for IFN β, which was significantly higher for Wb-12 compared to 03-03703 (Figure 4D). IFN α was only detected at low levels following infection of both viruses. The main IFN species generated under RV infection were IFN λ1 and λ2/3, which were induced at an almost comparable level by both RV strains, albeit for IFN λ2/3 at a significantly higher level under Wb-12 infection compared to 03-03703 (Figure 4D). These results were confirmed at the mRNA level for the main IFN types (IFN β, λ1 and λ2/3) induced during RV infection (Figure 4E). Already at 1 dpi a robust increase at the mRNA level was detected for type III IFNs for both strains. However, a significantly higher mRNA expression level was detected at 3 dpi for IFN λ2/3, which was comparable to the results by the LEGENDplex assay (Figure 4D). To determine the downstream effects of this IFN secretion, the mRNA expression level of selected IFN-stimulated genes (ISGs) was determined. The ISGs viperin and ISG15 were strongly induced by both viruses where only a moderate induction was detected for IFIT1, IFITM1 and IFITM3. While the increase in viperin mRNA levels was significantly higher under Wb-12 infection compared to 03-03703 (Figure 4F), all other ISGs were comparably induced by Wb-12 and 03-03703. Additionally, the IFN response under RV infection was comparable to poly I:C transfection, with the exception of the mRNA expression level of the ISG, IFIT1.

In conclusion, under RV infection the type III IFN response in A549 cells was more pronounced than type I. This results in the notable activation of the ISGs viperin and ISG15. While significant differences were noted for the activation of specific components of the IFN system, the overall activation of the IFN response was comparable among the RV strains examined.

### 3.5. RV Infection is Marginally Reduced after Pre-Treatment of A549 Cells with IFN β and Almost Insensitive to Type III IFNs

**Figure 5:**
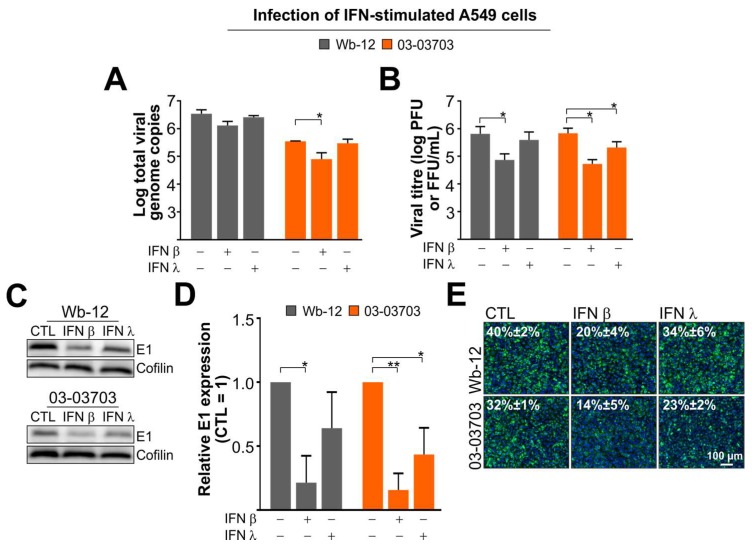
Stimulation of A549 cells with exogenous type I (IFN β) and III (IFN λ) IFNs affected RV infection. A549 cells were stimulated with IFN β or IFN λ (λ1, 2 and 3) for 72 h. Infection was compared to the untreated control (CTL) through assessment of (**A**) viral genome copies by TaqMan-based reverse transcription PCR and (**B**) titre determination by plaque and focus forming assay for samples collected at 1 dpi; (**C**) At 1 dpi E1 expression level in the absence or presence of exogenous IFN was determined with anti-E1 antibodies by Western blot analysis; (**D**) For assessment of relative E1 band intensities LI-COR image software was used. Indicated data is given as mean ±SE derived from three independent Western blot runs after normalization to cofilin; (**E**) Immunofluorescence analysis was performed at 1 dpi with anti-E1 antibodies (shown in green). Nuclei were stained with the DNA counterstain Hoechst bisbenzimide (shown in blue). Values in immunofluorescence images depict the mean percentage of RV-infected cells as determined by counting of the number of E1-positive cells in two random fields for two independent samples; (**C**,**E**) The images shown are representative for three independent experiments. *, *p* < 0.05; **, *p* < 0.01.

To determine if the produced IFNs played a role in restricting RV infection, A549 cells were pre-treated with IFN β or IFN λ (as a combination of λ1, 2 and 3) for 72 h. This time frame was chosen to mirror the long replication cycle of RV in cell culture. Additionally, effects observed after stimulation of A549 cells with exogenous IFNs for 12 or 24 h had only a minor effect as compared to pre-treatment for 72 h. RV infection and subsequent cultivation was carried out in the continued presence of the respective IFNs. As shown in Figure 5A, the pre-treatment of A549 cells with type I and III IFNs had only a minor effect on the intracellular amount of viral RNA. While the effect of IFN β was more pronounced than the one observed for IFN λ, a significant reduction was only noted for strain 03-03703. However, for both RV strains a significant reduction, one order of magnitude, in viral titre was detected in IFN β-pre-treated A549 cells (Figure 5B). A significant reduction was also noted after pre-treatment with IFN λ but only for 03-03703 strain. Accordingly, a reduction in viral E1 protein expression was noted for both RV strains through Western blot analysis (Figure 5C). Calculation of the relative band intensity of E1 protein obtained in three separate Western blots revealed for both RV strains a significant reduction after application of exogenous IFN β but the effect of exogenous IFN λ was only significant for 03-03703 strain (Figure 5D). Immunofluorescence analysis confirmed that 03-03703 was more sensitive to exogenous IFN λ than Wb-12 (Figure 5E). The level of significance for the reduction of the number of E1-positive cells after pre-treatment with IFN λ was higher for 03-03703 (*p* < 0.001) than for Wb-12 (*p* < 0.05).

Collectively these results suggest that although on A549 cells IFN λ was induced by RV at a greater degree than IFN β, a significant effect on RV infection was only noted for exogenous IFN β. Especially viral protein synthesis, as shown for E1, appeared to be affected by IFN pre-treatment. While the effect of IFN β on the different RV strains was comparable, the sensitivity against exogenous IFN λ differed slightly among RV strains.

### 3.6. An Equal Infection Rate of RV Strains on M1- and M2-Mφ Occurred in Association with a Strong Type I and III IFN Response

**Figure 6:**
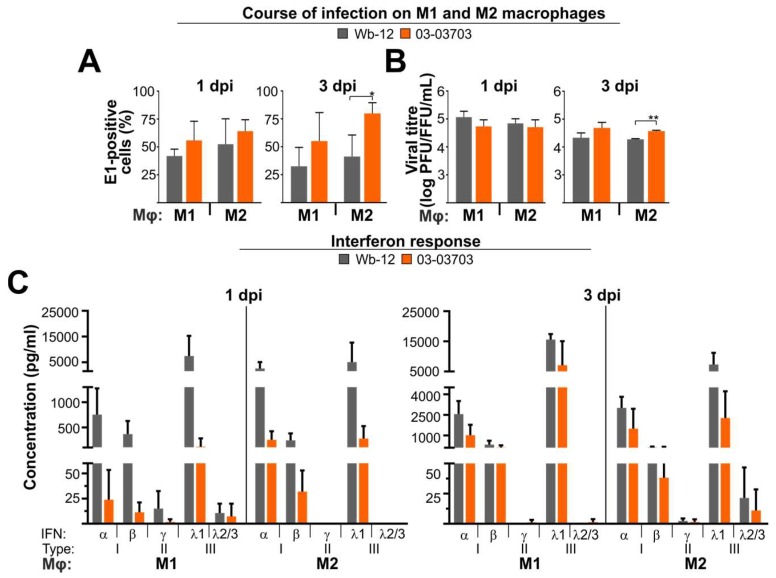
RV strains establish an equal infection rate on M1- and M2- macrophages (Mφ) and induce type I and III IFNs. (**A**) At 1 and 3 dpi RV-infected M1- and M2-Mφ were subjected to immunofluorescence analysis with an antibody against viral E1 protein. Percentage of E1-positive cells was determined for 5 independent microscopic fields obtained in three independent experiments. Supernatants collected at 1 and 3 dpi from M1- and M2-Mφ infected with indicated RV strains were collected and subjected to determination of (**B**) virus titre by plaque (PFU per mL) and focus-forming (FFU per mL) assay and (**C**) the type I/II/III IFN profile by the LEGENDplex IFN panel. *, *p* < 0.05; **, *p* < 0.01.

RV infection of M1- and M2-Mφ as human primary cells was carried out to determine if the IFN induction phenotype and sensitivity of A549 cells was also found in immune cells. We chose these cells as they are a promising cell type for analysis of RV infection, since granuloma M2-Mφ and alveolar Mφ in tissue samples of fatal CRS cases supported persistent RV infection [20,32]. Due to their central immunological functions they could play an important role in RV pathogenicity [33]. Furthermore, their high turn-over rate of glutamine could support replication of RV strains [10]. Mφ differentiated from monocytes derived from three different donors were infected with RV strains Wb-12 and 03-03703. In contrast to the lower infection rate of 03-03703 strain on Vero cells [10], the number of E1-positive cells during infection of M1- and M2-Mφ was comparable between the two strains at 1 dpi. Interestingly, at 3 dpi they were even significantly higher for 03-03703 as compared to Wb-12-infected M2-Mφ (Figure 6A). This was also reflected in viral titre, which was comparable at 1 dpi but at 3 dpi on M2-Mφ titre of 03-03703 was significantly higher compared to Wb-12 strain (Figure 6B). Furthermore, it is important to note, that the course of infection of both strains was comparable between the two Mφ types (Figure 6A,B). Supernatants collected at 1 and 3 dpi were subjected to analysis of IFN concentration by the LEGENDplex IFN panel. The IFN type and concentration was comparable between M1- and M2-Mφ (Figure 6C). On both Mφ types differences were noted at 1 dpi between the two RV strains in the overall concentration of IFNs. The IFN production was higher at 1 dpi on Wb-12-infected Mφ as compared to 03-03703, however they became comparable at 3 dpi (Figure 6C). Due to the variable response among the different samples examined for 1 dpi any noted differences between Wb-12 and 03-03703 were not significant. Similar to A549 cells, the type III IFN response was more pronounced than the one detected for type I. However, only IFN λ1 was generated on RV-infected Mφ (Figure 6C). Among type I IFNs, IFN α and β were generated at a similar concentration (Figure 6C). The ability of RV strains to establish high titre infection on M1- and M2-Mφ was also confirmed for Therien and 07-00426 strains (Supplement Figure S1A,B). Interestingly the 07-00426 strain showed a higher titre than Therien, which is in contrast to the Vero cell data, while IFN type and concentration of Therien and 07-00426 (Supplement Figure S1C) were comparable to Wb-12 to 03-03703 (Figure 6C). As a second primary human cell line, HUVEC were employed. The IFN profile of these cells was evaluated during infection with Wb-12 and 03-03703 (Supplement Figure S2A). Comparable to A549 cells, IFN β was the predominant type I IFN species and similar to Mφ IFN λ1 was detected as the predominant type III IFN. Finally, we examined the RV-associated IFN response on Vero, as a cell line with profound strain-specific differences in the infection rate [10]. Due to the deletion of type I IFN genes, only type III IFN response can be activated in Vero cells. Supplement Figure S2B highlights, that IFN λ1 was induced by RV strains Wb-12 and 03-03703, whereas IFN λ2/3 was lacking. While on A549 cells viperin and ISG15 were induced at a high rate, only ISG15 was found to be activated at a notable level on Vero cells. The remaining ISGs IFIT1, IFITM1 and IFITM3 were increased over the mock control at a reduced level. A significant difference in the induction of the ISGs between RV strains Wb-12 and 03-03703 was only detected for IFITM1 (Supplement Figure S2B).

The results on RV-infected Mφ in most parts mirror the ones on A549 cells in a RV strain-independent induction of an innate immune response, which is based on type I and III IFNs. Differences were noted with regard to the induced IFN species. In contrast to A549 cells, on Mφ IFN λ2/3 was almost lacking, while IFN α was present in addition to IFN β. This provides evidence that both Mφ types represent a suitable cell culture model for the analysis of innate immune response under RV infection and thus RV pathology.

## 4. Discussion

The elucidation of viral pathogenesis requires on the one hand low passaged clinical isolates to reflect diversity within the viral population. On the other hand, different human cell lines and various donors for primary human cells are needed as representatives for the genetic diversity found in the human population. This study revealed that while differences were noted for the induction of an oxidative stress response, the activation of an IFN response was comparable among RV strains. Moreover, it was emphasized that strain-specific differences in viral replication rate on Vero cells [10] are cell-type restricted, as human Mφ enabled a high level of replication irrespective of the applied RV strain. Additionally, the macrophage independent replication rate appears to be a unique property of RV, as differences are usually noted in the susceptibility of M1- and M2-Mφ to infections. The number of Lactococcus lactis, Escherichia coli, Leishmania major and human cytomegalovirus-infected, human monocyte-derived M2-Mφ was higher than those of M1-Mφ [22,34]. This was also reported for the course of infection of influenza virus on human bone marrow-derived M2-Mφ as compared to M1-Mφ [35] and of HIV-1 [36,37].

RV-induced alterations of mitochondrial functions might affect the activity of the electron transport chain and/or the mitochondrial membrane potential and could result in activation of an oxidative stress response. During RV infection, hyperpolarization of mitochondria [38] was noted in addition to an increased activity of respiratory chain complex II [11] and a higher bioenergetic profile of the infected host cell [10]. Due to this higher metabolic activity, the level of superoxide anion could increase, which in turn would be compensated by a higher expression level of SOD2. During RV infection SOD2 mRNA expression was increased and MitoSOX Red-sensitive superoxide was detected in mitochondria but more notably in the nucleus. Consistent with the finding that nuclear MitoSOX might be due to superoxide generation by a cytoplasmic ROS source or by superoxide released from mitochondria [39], cytoplasmic ROS could be induced through opening of the mitochondrial transition pore during RV infection [14]. Accordingly, strain-specific differences in metabolic alterations [10] were reflected by a differential induction of oxidative stress.

In contrast to the differential infection rate and activation of oxidative stress by RV strains, only minor differences were noted among RV strains with regard to the induction of an IFN response. The IFN response on Vero cells emphasizes that IFN activation appears not to be influenced by the differential replication kinetics displayed by RV strains on this cell line [10]. The activation of the IFN response and the associated induction of ISGs appear to be a common characteristic among RV strains. While such strain-specific effects were not detected for RV in our study, the induction of an innate immune response and accordingly its repression in A549 cells by respiratory syncytial virus was strain specific [40]. The non-structural PBI-F2 protein encoded by influenza A (IAV) enhances IFN β production and IAV strain-specific variants of this protein could contribute to viral immunopathology [41,42]. However, cell-type dependent differences for the IFN species generated during RV infection were noted in this study. An early study by Potter and colleagues reported notable differences in the induced levels of IFNs on placental but not on lung or leukocyte cell cultures by Japanese and US RV strains [43]. Similar to the Wb-12 strain, as highlighted by our study, the RV strain with the highest level of IFN induction had the lowest level of sensitivity against IFN [43].

The insensitivity of RV against exogenous IFN application and the cell type-specific differences in the activation of an IFN response could contribute to viral pathology and spread within its human host. Although treatment with IFN does not cure infected cultures from RV or viral replicons [6,44,45], it could contribute to the establishment of a persistent infection in IFN-competent cell lines as IFN attenuates RV cytopathogenicity [6]. Several viruses have developed countermeasures against innate immune responses as effective antiviral defence mechanisms. While Bluetongue virus interferes with IFN type I signalling and the JAK/STAT pathway [46], some viruses appear to be rather resistant to or only marginally affected by IFN-activated cellular response mechanisms. Besides RV as outlined in this study, hepatitis D virus (HDV) was recently described to replicate almost unaffected after activation of its host cell with exogenous IFNs [47]. This resistance was noted despite a notable induction of IFN β and IFN λ during HDV infection and was not based on viral interference with ISG expression [48]. In our study, RV replication occurred despite a profound activation of the ISGs viperin and ISG15. However, especially the application of exogenous IFN β revealed, that viral progeny release and protein synthesis were reduced while viral intracellular genomic RNA remained almost unaffected. This could reflect the protected environment provided by the cytopathic vacuoles that appear to shield RNA replication centres [49].

RV is a prominent example for distinct phenotypic and biological differences among virus strains [43]. These differences could reflect mechanisms of virus-host co-evolution and as such viral pathogenicity. It is noteworthy, that RV strains isolated from specimens after congenital infection display a higher rate of apoptosis as compared to attenuated strains [50]. Both, strain- and cell-type specific differences could contribute to our understanding of RV pathology. On the one hand RV causes mild and in most cases asymptomatic post-natal infections but on the other hand embryopathologies are extremely severe [51]. Further characterization of the strains highlighted in our study will address how the identified differences could be involved in the differential clinical outcome of a given virus infection. Human Mφ could be an important cell line for this approach, as Mφ cells enable solid replication of several RV strains at a similar rate and despite robust activation of an IFN response. Additionally, a more systematic analysis of RV strains on a cell culture model with relevance to RV teratogenicity is required. We have recently introduced induced pluripotent stem cells (iPSCs) as such a model [52]. They also represent a suitable in vitro model for RV persistence, which is established shortly after infection of the developing embryo and will be used to follow up the questions raised in this study.

## Supplementary Materials

The following are available online at http://www.mdpi.com/1999-4915/10/10/540/s1, Figure S1: Analysis of the time course of infection and immune activation of Therien and 07-00426 strains on M1- and M2-Mφ at 1 and 3 dpi, Figure S2: Characterization of the IFN response on RV-infected HUVEC and Vero cells.
