# Supplementary material for: Rubella Virus Strain-Associated Differences in the Induction of Oxidative Stress Are Independent of Their Interferon Activation
Source: Viruses. 2018 Oct 3;10(10):540. doi: 10.3390/v10100540 (PMC6213305; doi:10.3390/v10100540)
Supplement: Supplementary file 1 [file viruses-10-00540-s001.pdf]

*Supplementary information*

# Rubella Virus Strain-Associated Differences in the Induction of Oxidative Stress Are Independent of Their Interferon Activation

**Sarah Zobel [1,†], Mechthild Lorenz [1,†], Giada Frascaroli [2], Janik Böhnke [1,‡], Nicole C. Bilz [1], Megan L. Stanifer [3], Steeve Boulant [3,4], Sandra Bergs [1], Uwe G. Liebert [1] and Claudia Claus [1,*]**

[1] Institute of Virology, University of Leipzig, 04103 Leipzig, Germany; zobel.sarah@icloud.com (S.Z.); Mechthild.Lorenz@medizin.uni-leipzig.de (M.L.); janik.boehnke@gmail.com (J.B.); Christin.Emmrich@medizin.uni-leipzig.de (N.C.B.); Sandra.Bergs@medizin.uni-leipzig.de (S.B.); liebert@medizin.uni-leipzig.de (U.G.L.)

[2] Leibniz Institute for Experimental Virology, Heinrich Pette Institute, 20251 Hamburg, Germany; giada.frascaroli@leibniz-hpi.de

[3] Schaller Research Group at CellNetworks, Department of Infectious Diseases, Virology, Heidelberg University Hospital, 69120 Heidelberg, Germany; m.stanifer@dkfz-heidelberg.de (M.L.S.); s.boulant@Dkfz-Heidelberg.de (S.B.)

[4] Research Group "Cellular Polarity and Viral Infection" (F140), German Cancer Research Center (DKFZ), 69120 Heidelberg, Germany

* Correspondence: claudia.claus@medizin.uni-leipzig.de

‡ Current address: Institute for Biomedical Engineering, Department of Cell Biology, RWTH Aachen University, Medical School, 52074 Aachen, Germany.

† These authors contributed equally to this work.

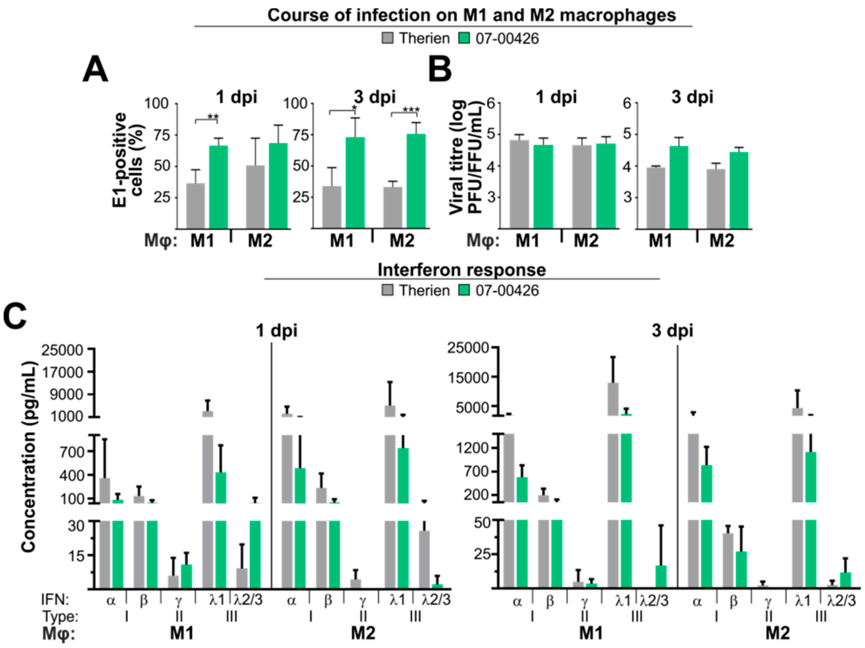


**Supplementary Figure S1**. Analysis of the time course of infection and immune activation of Therien and 07-00426 strains on M1- and M2-Mφ at 1 and 3 dpi. (A) The percentage of E1-positive cells was determined by immunofluorescence analysis with an antibody against viral E1 protein. Five independent microscopic fields were counted for three independent experiments. (**B**) Viral titre was determined by plaque and focus forming assay. (**C**) The type 1/2/3 IFN profile was determined by the LEGENDplex IFN panel.

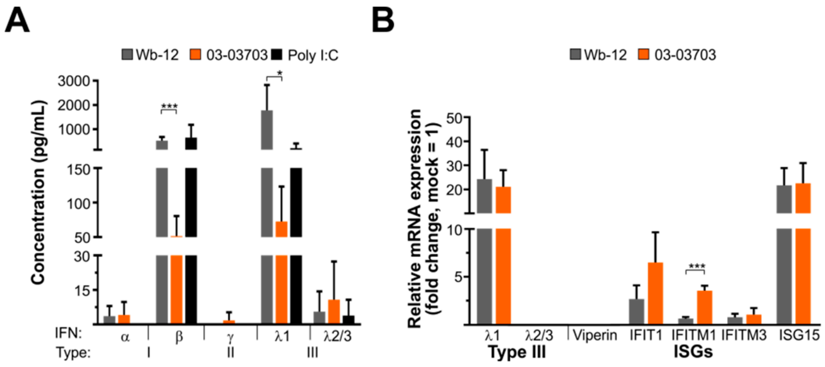


**Supplementary Figure S2**. Characterization of the IFN response on RV-infected HUVEC and Vero cells. (A) The type 1/2/3 IFN profile was determined at 36 hours post-infection (hpi) by the LEGENDplex IFN panel for HUVEC after infection with Wb-12 and 03-03703 strains. Supernatant collected at 4 hours post transfection from poly I:C-transfected HUVEC cells was used as a positive control. (B) The mRNA expression level of type III IFNs and indicated ISGs was determined at 3 dpi for Wb-12- and 03-03703-infected Vero cells by qRT-PCR.
